# Susceptibility of rheumatoid arthritis synovial fibroblasts to FasL- and TRAIL-induced apoptosis is cell cycle-dependent

**DOI:** 10.1186/ar2607

**Published:** 2009-02-05

**Authors:** Noreen Pundt, Marvin A Peters, Christina Wunrau, Simon Strietholt, Carsten Fehrmann, Katja Neugebauer, Christine Seyfert, Frans van Valen, Thomas Pap, Ingmar Meinecke

**Affiliations:** 1 Institute of Experimental Musculoskeletal Medicine, University Hospital Muenster, Domagkstr. 3, Muenster, 48149, Germany; 2 Institute of Medical Microbiology, University Hospital Muenster, Domagkstr. 10, Muenster, 48149, Germany; 3 Department of Orthopaedic Surgery, Zeisigwaldkliniken Bethanien Chemnitz, Zeisigwaldstr. 101, Chemnitz, 09130, Germany; 4 Department of Orthopaedic Surgery, Park-Krankenhaus Leipzig-Suedost GmBH, Struempellstr. 41, Leipzig, 04289, Germany

## Abstract

**Introduction:**

The rheumatoid arthritis (RA) synovium is characterised by the presence of an aggressive population of activated synovial fibroblasts (RASFs) that are prominently involved in the destruction of articular cartilage and bone. Accumulating evidence suggests that RASFs are relatively resistant to Fas-ligand (FasL)-induced apoptosis, but the data concerning tumour necrosis factor-related apoptosis-inducing ligand (TRAIL) have been conflicting. Here, we hypothesise that the susceptibility of RASFs to receptor-mediated apoptosis depends on the proliferation status of these cells and therefore analysed the cell cycle dependency of FasL- and TRAIL-induced programmed cell death of RASFs *in vitro*.

**Methods:**

Synovial fibroblasts were isolated from patients with RA by enzymatic digestion and cultured under standard conditions. Cell cycle analysis was performed using flow cytometry and staining with propidium iodide. RASFs were synchronised or arrested in various phases of the cell cycle with 0.5 mM hydroxyurea or 2.5 μg/ml nocodazol and with foetal calf serum-free insulin-transferrin-sodium selenite supplemented medium. Apoptosis was induced by stimulation with 100 ng/ml FasL or 100 ng/ml TRAIL over 18 hours. The apoptotic response was measured using the Apo-ONE^® ^Homogenous Caspase-3/7 Assay (Promega GmbH, Mannheim, Germany) and the Cell Death Detection (ELISA^Plus^) (enzyme-linked immunosorbent assay) (Roche Diagnostics GmbH, Mannheim, Germany). Staurosporin-treated cells (1 μg/ml) served as a positive control. Expression of Fas and TRAIL receptors (TRAILR1-4) was determined by fluorescence-activated cell sorting analysis.

**Results:**

Freshly isolated RASFs showed only low proliferation *in vitro*, and the rate decreased further over time, particularly when RASFs became confluent. RASFs expressed Fas, TRAIL receptor-1, and TRAIL receptor-2, and the expression levels were independent of the cell cycle. However, the proliferation rate significantly influenced the susceptibility to FasL- and TRAIL-induced apoptosis. Specifically, proliferating RASFs were less sensitive to FasL- and TRAIL-induced apoptosis than RASFs with a decreased proliferation rate. Furthermore, RASFs that were synchronised in S phase or G_2_/M phase were less sensitive to TRAIL-induced apoptosis than synchronised RASFs in G_0_/G_1 _phase.

**Conclusions:**

Our data indicate that the susceptibility of RASFs to FasL- and TRAIL-induced apoptosis depends on the cell cycle. These results may explain some conflicting data on the ability of RASFs to undergo FasL- and TRAIL-mediated cell death and suggest that strategies to sensitise RASFs to apoptosis may include the targeting of cell cycle-regulating genes.

## Introduction

Rheumatoid arthritis (RA), a chronic disease of incompletely understood aetiology, is characterised primarily by the progressive destruction of articular structures. Its pathogenesis is governed by the concerted action of several cell types that create signs and symptoms characteristic for RA. Accumulating evidence indicates that, in addition to macrophages and T cells, activated RA synovial fibroblasts (RASFs) play a major role in both initiating and driving the disease [1-4]. Not only do RASFs with an aggressive phenotype increase in number, their activation also results in the production of proinflammatory mediators and matrix-degrading enzymes and in alterations of programmed cell death [3-5].

Programmed cell death, or apoptosis, is central for both development and tissue homeostasis of metazoans. Therefore, aberrations of this process may lead to a variety of human pathologies, including cancer, autoimmune diseases, and neurodegenerative disorders. Apoptosis can be induced by members of the tumour necrosis factor (TNF) receptor family through the recruitment of an intracellular membrane-associated complex of proteins (death-inducing signaling complexes, or DISCs), which leads to a cytoplasmic release of active caspase-8 and subsequent activation of the apoptotic cascade [6,7]. Among these death receptors, Fas/CD95 and its specific ligand FasL/CD95L were demonstrated to be of importance, and it was shown that stimulation of RASFs with FasL initiates proapoptotic signals [8,9]. However, several studies with cultured RASFs showed that stimulation of RASFs with Fas-activating ligands induced apoptosis in only a small percentage of cells, and several mechanisms have been identified that prevent RASFs from Fas-mediated cell death [10-16]. Actually, several studies have shown that RASFs undergo less FasL-induced apoptosis than osteoarthritis synovial fibroblasts and therefore RASFs has been termed relatively resistant to FasL-induced apoptosis. As shown previously, fibroblasts in RA synovium express both TNF-α receptors and Fas, and their ligands have been detected in co-localised macrophages and T cells [17-19].

TNF-related apoptosis-inducing ligand (TRAIL), another member of the TNF superfamily of apoptosis-inducing ligands, can bind to five receptors. Among them, TRAIL-R3 (DcR1) and TRAIL-R4 (DcR2) act as membrane-anchored decoy receptors, whereas TRAIL-R1 (DR4) and TRAIL-R2 (DR5) contain a cytoplasmic death domain and transmit proapoptotic signals into cells [20]. In addition, osteoprotegerin, a soluble decoy receptor of the ligand for the receptor activator of nuclear factor-kappa-B (NF-κB) (RANKL), has been shown to bind TRAIL [21,22]. Apoptosis can be induced upon binding of TRAIL to DR4 and DR5 and subsequent activation of different caspases. On the other hand, studies suggest that binding of TRAIL to these receptors can also induce proliferation through activation of the NF-κB signalling pathway [23,24], and it appears that the ability of TRAIL to trigger either apoptosis or cell survival depends on the cell type [25].

The *in vitro *data concerning TRAIL-induced apoptosis in RASFs have been conflicting. Morel and colleagues [25] showed that exposure to TRAIL induced apoptosis in only 30% of RASFs within 24 hours whereas surviving cells proliferated in a TRAIL dose-dependent manner. In contrast, Ichikawa and colleagues [26] documented TRAIL (anti-DR5 antibody)-induced apoptosis of RA synovial cells with 80% of the cells being killed. In both studies, RASFs showed constitutive expression of TRAIL receptor-2 (DR5) as the main mediator of TRAIL-induced stimulation. In addition, Morel and colleagues [25] could show the expression of TRAIL-R1 (DR4). Here, we hypothesise that the susceptibility of RASFs to receptor-mediated apoptosis depends on the proliferation state of these cells. Therefore, we analysed the cell cycle dependency of FasL- and TRAIL-induced programmed cell death of RASFs *in vitro*.

## Materials and methods

### Patients and tissue samples

Samples of synovial membrane from patients with RA (according to the 1987 revised American College of Rheumatology criteria) were obtained at joint replacement surgery within an ongoing national tissue bank project with the 'Assoziation für Rheumatologische Orthopädie' (ARO) of the German Society of Rheumatology (DGRh) and provided by the Department of Orthopaedic Surgery of St. Joseph Hospital (Sendenhorst, Germany), the Department of Orthopaedic Surgery of the University of Magdeburg School of Medicine (Magdeburg, Germany), and the Department of Orthopaedic Surgery (KMG-Kliniken Kyritz, Germany). Approval from the local ethics committee was obtained prior to starting the study. Fibroblasts were isolated by digesting synovial tissue with 1.5 mg/ml Dispase II (Roche Diagnostics GmbH, Mannheim, Germany) and cultured in complete Dulbecco's modified Eagle's medium (DMEM supplemented with 10% foetal calf serum [FCS], Invitrogen Corporation, Carlsbad, CA, USA, and penicillin/streptomycin, PAA, Pasching, Austria) as described previously [27]. Fibroblasts were used in passages 4 to 8.

### Fluorescence-activated cell sorting analysis

Flow cytometric analysis of cell cycle was performed as described previously [28]. Briefly, cells were detached with 1 mM ethylenediaminetetraacetic acid (EDTA) and suspended in fluorescence-activated cell sorting (FACS) buffer (phosphate-buffered saline [PBS] supplemented with 5% FCS and 0.1% NaN_3_). Cell cycle analysis was performed by incubation of cells with propidium iodide (40 μg/ml propidium iodide, 100 μg/ml RNase in PBS) for up to 2 days and subsequent flow cytometry (FACScalibur; BD Biosciences, San Jose, CA, USA). To arrest RASFs in G_2_/M phase, cells were treated with nocodazol (2.5 μg/ml in DMEM for 18, 24, or 36 hours; Calbiochem, Darmstadt, Germany). Furthermore, randomly growing cultures of RASFs were synchronised with 0.5 mM hydroxyurea (HU) (Sigma-Aldrich, Steinheim, Germany) in DMEM and incubated at 37°C for 6 hours. Cells were washed with PBS and suspended in fresh complete DMEM. Synchronised RASFs were incubated at 37°C and samples (0, 18, 24, 30, 42, and 48 hours) thereof were analysed for cell cycle by propidium iodide staining as described above. In addition, RASFs were arrested in G_0_/G_1 _phase by serum deprivation. To this end, cultures of RASFs were incubated with DMEM supplemented with 1× insulin-transferrin-sodium selenite (ITS) supplement (100×) (Sigma-Aldrich) [29,30] for up to 10 days (0, 3, 8, and 10 days) following incubation with complete medium for 1 or 2 days (9/1, 9/2 days).

### Analysis of Fas- and TRAIL-receptor expression

Surface expression of Fas and TRAIL receptors (TRAILR1-4) on RASFs was determined by flow cytometry as described [31]. Briefly, 1 × 10^5 ^cells were labelled with 0.5 μg of mouse anti-TRAILR1-4 (Alexis Biochemicals, Lörrach, Germany), mouse anti-Fas antibodies, or mouse anti-IgG in FACS buffer containing 5 mM EDTA for 40 minutes at 4°C. These cells were incubated with biotin-conjugated goat anti-mouse, phycoerythrin-conjugated anti-goat, or fluorescein isothiocyanate-conjugated anti-mouse antisera for 30 minutes at 4°C. Stained cells were fixed and 1 × 10^4 ^viable cells were analysed by flow cytometry using standard settings.

### Induction and measurement of apoptosis

Apoptosis was induced at different density states or cell cycle phases by incubation of cells with 100 ng/ml FasL (Bender MedSystems, Vienna, Austria) or 100 ng/ml TRAIL (Pepro Tech, Rocky Hill, NJ, USA) in 100 μL of complete DMEM or DMEM for 18 hours. The apoptotic response was measured by Cell Death Detection (ELISA^Plus^) (enzyme-linked immunosorbent assay) (Roche Diagnostics GmbH) and the Apo-ONE^® ^Homogeneous Caspase-3/7 Assay (Promega GmbH, Mannheim, Germany) in accordance with the instructions of the manufacturer. Staurosporin-treated cells (1 μg/ml, 8 hours) served as a positive control.

### Statistical analysis

Data shown are mean ± standard deviation. Statistical analysis was performed using GraphPad Prism Software version 4.0 (GraphPad Software Inc., San Diego, CA, USA). Differences between groups were examined for statistical significance using the Mann-Whitney test, and a *P *value of less than 0.05 was considered statistically significant.

## Results

### Proliferation of rheumatoid arthritis synovial fibroblasts

**Figure 1:**
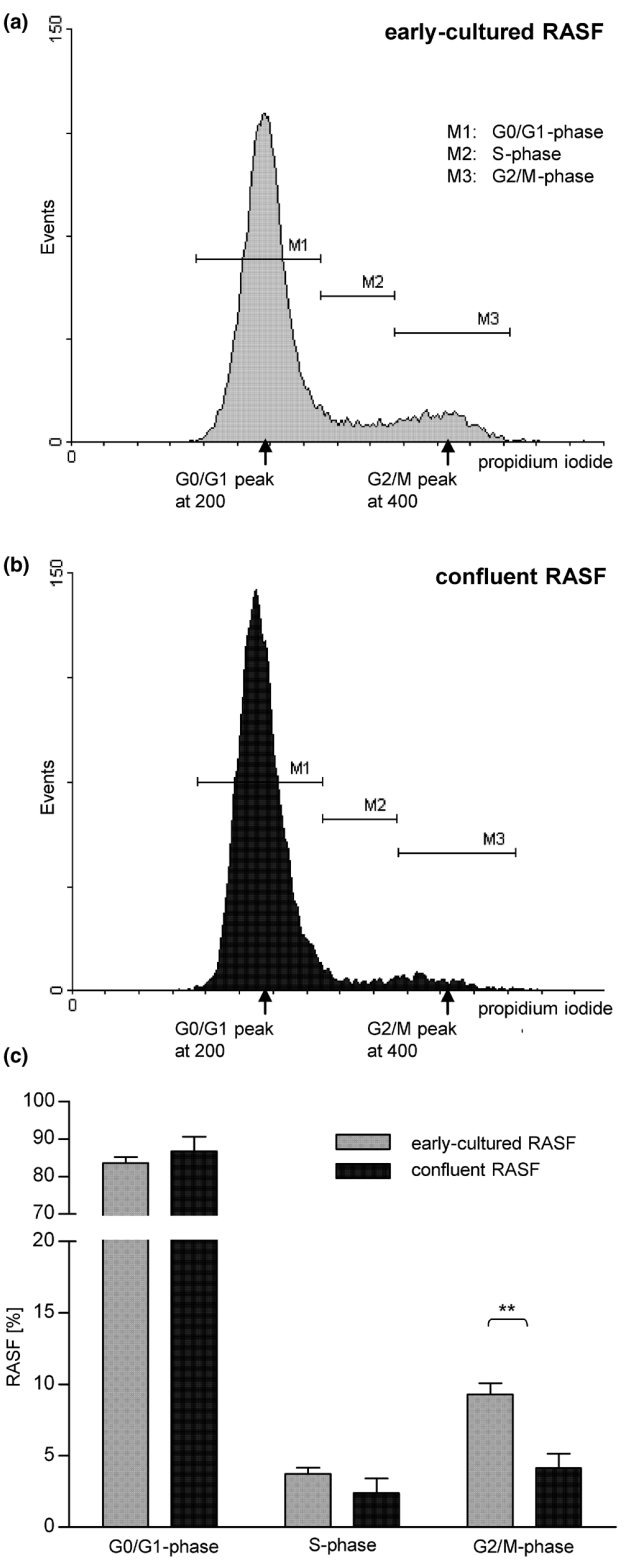
Proliferation capacity of rheumatoid arthritis synovial fibroblasts (RASFs). **(a) **Early-cultured RASFs exhibit only a very low proliferation rate *in vitro *(>2 n DNA equates S phase and G_2_/M phase, representative histogram). ~2 n DNA (arrow at 200) refers to the normal DNA content of interphase (G_0_/G_1 _phase) RASFs [32]. 4 n DNA (arrow at G_2_/M peak at 400) refers to twice the amount of DNA in G_2_/M compared with G_0_/G_1 _phase. **(b) **Decrease in proliferation rate in confluent RASFs. **(c) **Quantitative analysis. Values are mean ± standard deviation as a percentage of early-cultured and confluent RASFs obtained from 11 or 6 individual patients with rheumatoid arthritis. **P < 0.01.

**Figure 2:**
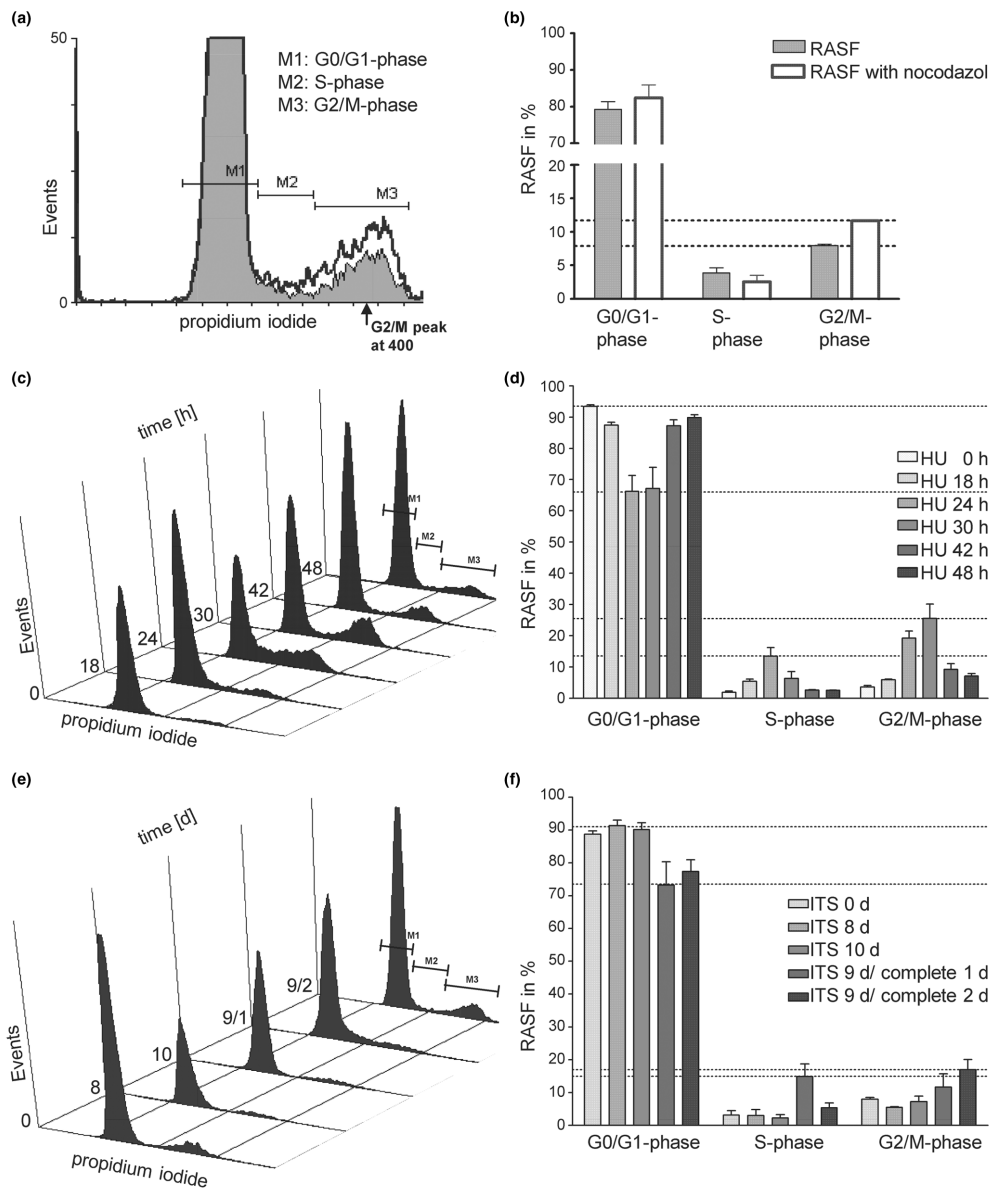
Effects of synchronisation and cell cycle arrest on proliferation of rheumatoid arthritis synovial fibroblasts (RASFs). **(a) **The effect of nocodazol on the cell cycle of early-cultured RASFs. Treatment of higher-proliferating RASFs with nocodazol (2.5 μg/ml, 18 hours) resulted in a marginal increase of RASFs arrested in G_2_/M phase (~4 n DNA at G_2_/M peak, black line, representative histogram). **(b) **Quantitative analysis. Values are mean ± standard deviation as a percentage of RASFs treated/untreated with nocodazol obtained from 3 individual patients with RA. **(c) **Effects of hydroxyurea (HU) on cell cycle of RASFs were illustrated in a representative three-dimensional histogram with the y-axis (time in hours) pointing away from the observer. RASFs treated with HU for 6 hours (time 0 hours) showed an accumulation of RASFs in G_0_/G_1 _phase. Analysis of cell cycle 18, 24, and 30 hours after HU exposure showed a decrease of RASFs in G_0_/G_1 _phase with a simultaneous increase of proliferating RASFs in S phase and G_2_/M phase, indicating that the cell population remained highly synchronised. Cell cycle analysis after 42 and 48 hours confirmed an increase of RASFs in G_0_/G_1 _phase, indicating that cell division commenced between 30 and 48 hours. **(d) **Quantitative analysis as mean ± standard deviation. **(e) **Early-cultured RASFs became arrested at G_0_/G_1 _phase after 8 to 10 days of incubation with ITS medium. Subsequent incubation for another 1 or 2 days with complete Dulbecco's modified Eagle's medium (9/1, 9/2 days) resulted in an increase of proliferating RASFs. Bar graphs in frames on right show quantitative analysis. Values are presented as mean ± standard deviation of percentages of RASFs obtained from at least three individual patients with rheumatoid arthritis. Representative three-dimensional histogram. **(f) **Quantitative analysis.

First, we analysed DNA content by FACS analysis to determine the proliferation rate of RASFs. Early-cultured RASFs exhibited a proliferation rate of 13.01%, according to cells with a DNA content of greater than 2 n (Figure 1a, representative histogram, and Figure 1c, DNA content in S and G_2_/M phases, n = 11). ~2 n DNA refers to the normal DNA content in the interphase (G_0_/G_1 _phase, diploid) of RASFs [32]. Confluent RASFs (100% confluent, 10^4 ^cells) exhibited a proliferation rate of 6.53% (Figure 1b, representative histogram, and Figure 1c, n = 5), significantly lower compared with early-cultured RASFs (Figure 1c, *P *= 0.0028). Nocodazol, the microtubule-destabilising agent that disrupts spindle assembly and impedes re-entry into the cell cycle [32,33], was used to arrest RASFs at G_2_/M phase (~4 n DNA). Cell cycle analysis of early-cultured RASFs (10^4 ^cells) treated with nocodazol for 18 hours showed only a marginal increase of proliferating RASFs to G_2_/M phase, from 7.95% to 11.41%, corresponding to ~4 n DNA content (Figure 2a, representative histogram, and Figure 2b, n = 5). Similar results were obtained after incubation with nocodazol for 24 and 36 hours (data not shown). MHH-ES-1 cells, an established Ewing sarcoma cell line [34], were used as a positive control for arresting cells in G_2_/M phase after incubation with nocodazol. G_2_/M-phase-arrested MHH-ES-1 cells showed a 20% increase in the G_2_/M phase, from 46% to 66% (data not shown). HU, which inhibits reversible DNA synthesis in mammalian cells without affecting RNA and protein synthesis, was used to synchronise RASFs in G_0_/G_1 _phase [35]. The effect of HU on the cell cycle of RASFs was illustrated in Figure 2c (representative histogram) and Figure 2d (n = 3). Cell cycle analysis of RASFs treated with a single exposure to 0.5 mM HU for 6 hours (time 0 hours) showed an accumulation of RASFs in G_0_/G_1 _phase (93.39%, corresponding to ~2 n DNA, n = 3), indicating that the cell population remained highly synchronised. Figure 2c and 2d also illustrated the cell cycle of RASFs after various hours after reversal of HU. Analysis of cell cycle 18, 24, 30, 42, and 48 hours after HU exposure showed a decrease of RASFs in G_0_/G_1 _phase until 66.24% (-27.15%, after 24 hours, n = 3) with simultaneous increase of proliferating RASFs in S phase, reaching a maximum at 24 hours (+11.55%, n = 3), and G_2_/M phase, reaching a maximum at 30 hours (+25.53%, ~4 n DNA, n = 3). Forty-two hours after HU exposure, cell cycle analysis confirmed an increase of RASFs in G_0_/G_1 _phase back to 87.18%, and after 48 hours to 89.83%, indicating that cell division commenced between 30 and 48 hours. No higher degree of synchronisation was induced by a subsequent second exposure to HU (data not shown). In addition, RASFs were arrested in G_0_/G_1 _phase through serum deprivation using ITS supplement. As illustrated in Figure 2e (representative histogram) and Figure 2f (n = 3), early-cultured RASFs became arrested at G_0_/G_1 _phase after 8 to 10 days of incubation with ITS medium. The initial rate of proliferating RASFs decreased from 11.14% to 8.56%, or 7.96% (corresponding to <2 n DNA, from 0 d to 8 d, and 10 d, n = 3). Subsequent incubation for another one or two days with complete DMEM resulted in an increase of proliferating RASFs to 25.95% (<2 n DNA, 9 days of ITS medium/1 day of complete medium, 9/1 d) or 22.34% (9/2 d). Maximum of RASFs in S phase was reached at day 9/1 (+12.02%, n = 3) and in G_2_/M phase at day 9/2 (+11.3%, n = 3). These results suggest that only a small population of early-cultured RASFs proliferate.

### Susceptibility of rheumatoid arthritis synovial fibroblasts to FasL- and TRAIL-induced apoptosis

**Figure 3:**
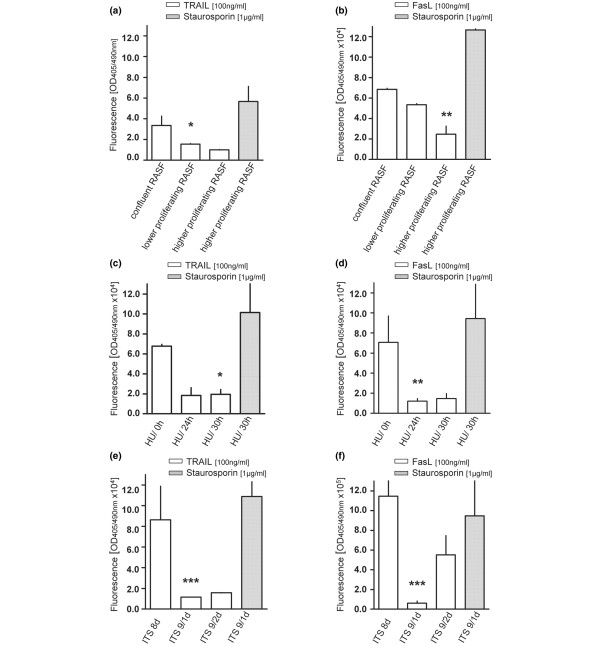
Susceptibility of proliferating rheumatoid arthritis synovial fibroblasts (RASFs) to Fas ligand (FasL)-induced and tumour necrosis factor-related apoptosis-inducing ligand (TRAIL)-induced apoptosis. **(a) **As assessed by Cell Death Detection (ELISA^Plus^), higher-proliferating RASFs (50% of confluency) were less sensitive to TRAIL-induced apoptosis than lower-proliferating RASFs (80% of confluency) and significantly less sensitive than confluent RASFs (100% confluent). **(b) **As revealed by the Apo-ONE^® ^Homogeneous Caspase-3/7 Assay, higher-proliferating RASFs showed lower activities of caspase-3 and caspase-7 after induction of apoptosis with FasL than less-proliferating RASFs and confluent RASFs. Highly synchronised RASFs in S phase (HU/24 h) or G_2_/M phase (HU/30 h) were less sensitive to TRAIL-induced **(c) **and FasL-induced **(d) **apoptosis than synchronised RASFs in G_0_/G_1 _phase (HU/0 h), as measured by the Apo-ONE^® ^Homogeneous Caspase-3/7 Assay. Moreover, RASFs arrested in G_0_/G_1 _phase through serum deprivation using insulin-transferrin-sodium selenite (ITS) medium (8 d) were more sensitive to TRAIL-induced **(e) **and FasL-induced **(f) **apoptosis than proliferating RASFs in S phase (9/1 d) or G_2_/M phase (9/2 d). Staurosporin-induced apoptosis was measured as a positive control. All values are mean ± standard deviation of fluorescence/fluorescence of unstimulated RASFs from at least three independent patients with rheumatoid arthritis. *P < 0.05, **P < 0.01, ***P < 0.001.

Next, we analysed the cell cycle dependency of FasL- and TRAIL-induced programmed cell death of RASFs *in vitro*. We found that higher-proliferating RASFs (50% of confluency) from different patients were less sensitive to TRAIL-induced apoptosis than lower-proliferating RASFs (80% of confluency) and even significantly less sensitive when confluent RASFs (100% confluent) were used as measured by Cell Death Detection (ELISA^Plus^). As Figure 3a illustrates, the photometric enzyme immunoassay for the detection of cytoplasmic histone-associated DNA fragments showed a reduction from 3.35 relative fluorescence units (RFU) (confluent RASFs) to 1.55 RFU (-53%, lower-proliferating RASFs) or to 1.0 RFU (-70.15%, higher-proliferating RASFs, data are presented as optical density (OD)/OD untreated RASFs, n = 7). Similar observations were made when RASFs in different density states were treated with FasL. Measurement of the activities of caspase-3 and caspase-7, key effectors of apoptosis in mammalian cells, revealed that higher-proliferating RASFs (50% of confluency) were less sensitive to FasL-induced apoptosis than lower-proliferating RASFs (80% of confluency) and confluent RASFs (Figure 3b). A reduction from 6.79 × 10^4 ^RFU (confluent RASFs) to 5.26 × 10^4 ^RFU (-22.5%, lower-proliferating RASFs) and to 2.8 × 10^4 ^RFU (-59%, higher-proliferating RASFs, n = 3) was observed. Furthermore, highly synchronised RASFs in S phase (HU, time 24 hours, Figure 2c,d) and in G_2_/M phase (time 30 hours) were less sensitive to TRAIL-induced apoptosis than synchronised RASFs in G_0_/G_1 _phase (time 0 hours, Figure 3c). A reduction from 4.84 × 10^4 ^RFU (HU/0 hours, n = 5) to 1.83 × 10^4 ^RFU (-62.2%, HU/24 hours, n = 5) or to 1.93 × 10^4 ^RFU (-60.13%, HU/30 hours, n = 5) was observed by measurement of the activities of caspase-3 and caspase-7. Similar results were obtained after measurement of FasL-induced apoptosis. Compared with RASFs synchronised in G_0_/G_1 _phase (7.06 × 10^4 ^RFU, n = 3, Figure 3d), RASFs synchronised in S phase showed a reduced apoptotic response of 1.2 × 10^4 ^RFU (-83.01%, n = 3) and RASFs synchronised in G_2_/M phase showed a reduced apoptotic response of 1.45 × 10^4 ^RFU (-79.5%, n = 3). Moreover, RASFs arrested in G_0_/G_1 _phase through serum deprivation using ITS medium (8 d) were more sensitive to TRAIL- and FasL-induced apoptosis than proliferating RASFs in S phase (9/1 d) or in G_2_/M phase (9/2 d, Figure 3e,f). TRAIL-induced caspase-3/7 activities decreased from 8.62 × 10^4 ^RFU in RASFs arrested in G_0_/G_1 _phase to 1.15 × 10^4 ^RFU (-86.6%, n = 3) in RASFs arrested in S phase and to 1.54 × 10^4 ^RFU (-82.1%, n = 3) in RASFs arrested in G_2_/M phase. Again, comparable results were obtained by measurement of FasL-induced programmed cell death. Figure 3f illustrates a reduction from 1.14 × 10^6 ^RFU (G_0_/G_1 _phase, 8 d) to 0.61 × 10^5 ^RFU (-94.64%, S phase, 9/1 d) and to 5.52 × 10^5 ^RFU (-51.84%, G_2_/M phase, 9/2 d). Unless otherwise noted, all data in Figure 3 are presented as OD/OD unstimulated RASFs. Staurosporin-treated cells served as a positive control. We hypothesise that the susceptibility of RASFs to receptor-mediated apoptosis depends on the proliferation state of these cells *in vitro*.

### Expression of Fas and TRAIL receptors on rheumatoid arthritis synovial fibroblasts

**Figure 4:**
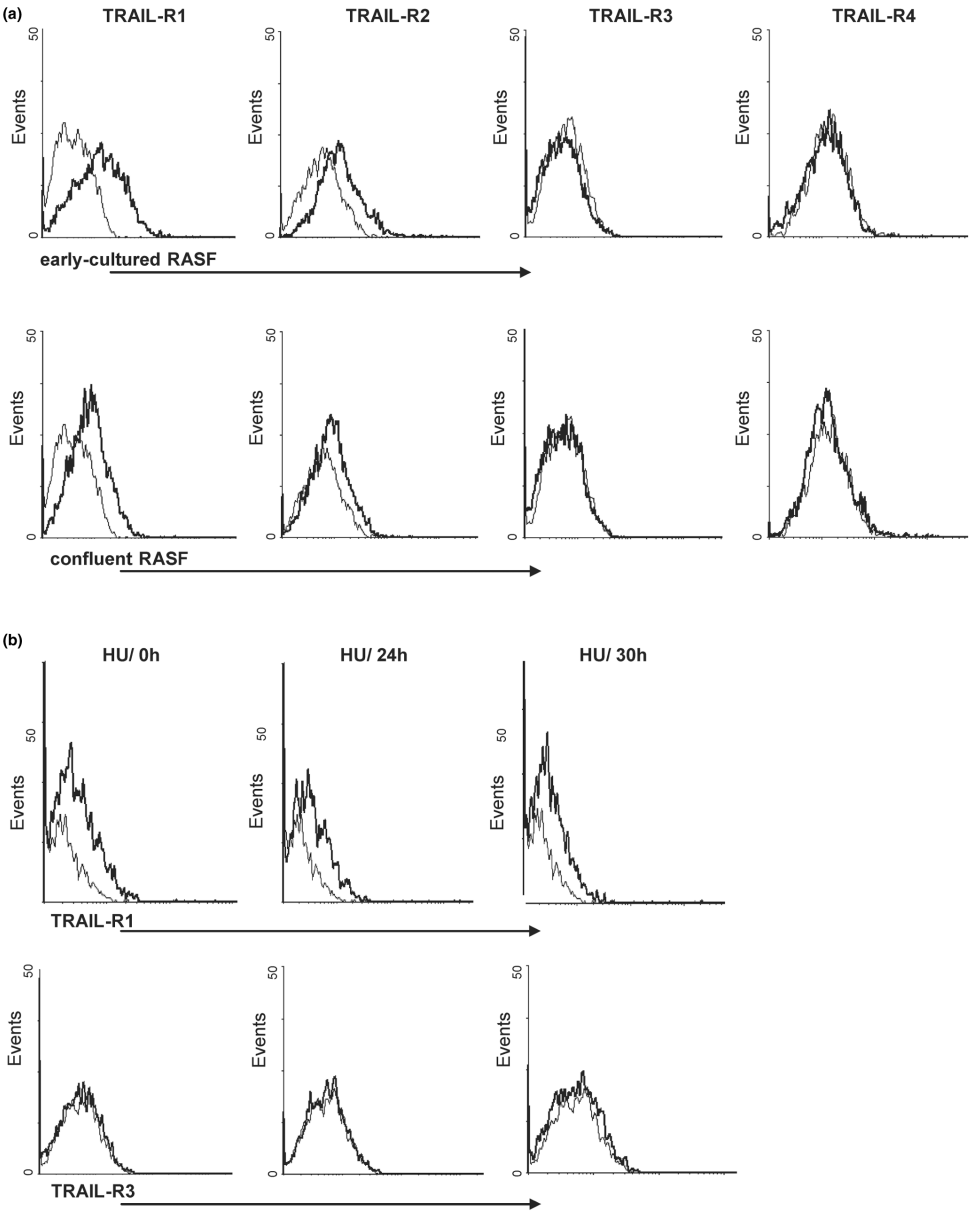
Surface expression of tumour necrosis factor-related apoptosis-inducing ligand (TRAIL) receptors on rheumatoid arthritis synovial fibroblasts (RASFs). **(a) **Staining of TRAIL receptors, as analysed by flow cytometry, showed constitutive surface expression of TRAIL-R1 and TRAIL-R2 on RASFs *in vitro*. TRAIL-R3 and TRAIL-R4 were not detectable. The expression levels did not change in confluent RASFs. **(b) **Furthermore, cell surface expression of TRAIL-R1 and TRAIL-R3 on RASFs remained unchanged in RASFs synchronised with hydroxyurea (HU). Representative histograms of three separate experiments are shown.

Finally, to investigate whether altered expression of death receptors may provide an explanation for differences in the susceptibility of RASFs to FasL- and TRAIL-induced apoptosis, the expression of Fas- and TRAIL-receptor changes during cell cycle progression, synchronisation, or at cell cycle arrest was examined. As shown by flow cytometry, TRAIL-R1 and TRAIL-R2 were expressed constitutively on higher-proliferating RASFs *in vitro*, whereas TRAIL-R3 and TRAIL-R4 were not detectable. The expression levels did not change in confluent RASFs (Figure 4a, representative histogram, n = 3). In addition, expression of these receptors was unaltered when RASFs were treated for 18 hours with 100 ng/ml TRAIL (data not shown). Furthermore, cell surface expression of TRAIL receptors on RASFs remained unchanged in RASFs synchronised with HU (Figure 4b, representative histogram, n = 3) or on RASFs arrested by using ITS medium (data not shown).

Fas (CD95) is a well-known apoptosis-signalling cell surface receptor belonging to the TNF receptor family [36]. To investigate the susceptibility of RASFs to FasL-mediated apoptosis, cell surface expression of Fas on RASFs was determined by flow cytometry *in vitro*. In agreement with data from Kobayashi and colleagues [37], who showed surface expression of Fas on RA synoviocytes, Fas was constitutively expressed on higher-proliferating RASFs (data not shown). Cell surface expression remained unchanged in confluent RASFs and under all investigated conditions (data not shown).

## Discussion

A decreased susceptibility to apoptosis and synovial proliferation has been described to contribute to RASF hyperplasia [5,10,11,14,38,39]. In this context, the TRAIL receptor/TRAIL system and the Fas/FasL system have raised much interest. Increasing evidence suggests that RASFs are relatively resistant to FasL-induced apoptosis *in vitro *[10,11,40]. Specifically, several studies with cultured RASFs showed that synoviocytes from rheumatoid synovium tissue express functional Fas [8,17,18] and that Fas activation induces apoptosis only in a small population of cells, even though the Fas/FasL system seems to be incapable of eliminating cells in proliferative RA synovium [8,18,37,40,41]. The data concerning TRAIL appear to be controversial [25,26,40]. Ichikawa and colleagues [26] analysed the effect of TRAIL on RASFs and reported an increased DR5 expression and an induction of DR5-mediated apoptosis up to 80%, although varying levels of apoptosis were induced by TRAIL using different RASF cultures. In agreement with these findings, Miranda-Carus and colleagues [38] analysed fibroblasts of 50 RA synovial fluid samples and showed that these fibroblasts underwent apoptosis when treated *in vitro *with an agonistic anti-DR5 antibody. In contrast, Morel and colleagues [25] proposed that TRAIL might have two different effects on RASFs, namely an initial rapid induction of apoptosis of up to 30% within the first 24 hours followed by an increase in the proliferation [25]. In addition, it is well documented that, depending on the cellular system, TRAIL can promote both proliferation and apoptosis, as has been established for other members of the TNF cytokine family [42]. In the present study, we hypothesised that the susceptibility of RASFs to receptor-mediated apoptosis depends on the proliferation status of these cells and, therefore, analysed the cell cycle dependency of FasL- and TRAIL-induced programmed cell death of RASFs *in vitro*.

Our results indicate that freshly prepared RASFs exhibit only a very low proliferation rate *in vitro*. The proliferation rate decreases further over time, particularly when RASFs become confluent. Furthermore, we describe for the first time that up to 65% of RASFs exhibit a G_0_/G_1_-phase arrest *in vitro*. Moreover, our study shows that early-cultured RASFs are less sensitive to TRAIL- and FasL-induced apoptosis than late-cultured RASFs and far less sensitive than 100% confluent RASFs. The difference in sensitivity to TRAIL- and FasL-mediated apoptosis between early-cultured and confluent RASFs is not due to differences in the surface expression of Fas and TRAIL receptors. Rather, the susceptibility clearly depended on the cell cycle of these cells as RASFs that were synchronised in S phase or G_2_/M phase were less sensitive to TRAIL-induced apoptosis than RASFs that were arrested in G_0_/G_1 _phase. These results suggest an inverse correlation between cell proliferation and apoptosis. However, how the proliferation influences TRAIL- and FasL-mediated synovial cell death remains unclear. Miyashita and colleagues [43] proposed that the serine/threonine protein kinase Akt, which affects several important cellular functions (including cell growth, cell cycle entry, migration, and cell survival), is an endogenous inhibitor of the TRAIL-mediated synovial cell apoptotic pathway. Furthermore, numerous data have shown that activation of Akt inhibits TRAIL-mediated apoptosis in various cancer cells and Akt has been shown to be overexpressed and activated in rheumatoid synovial cells *in situ *[44-47]. Therefore, it might be speculated that there is a correlation between cell proliferation and apoptosis, which may be regulated by the Akt pathway, but clearly further studies are required to elaborate on these observations.

## Conclusion

In summary, we have shown that a relatively high number of RASFs are arrested in G_0_/G_1 _phase. Furthermore, our data indicate that the sensitivity to TRAIL- or FasL-mediated apoptosis may be closely linked to synovial proliferation. These findings will further enhance our understanding of the pathophysiology of RA.

## Abbreviations

2 n DNA: diploid chromosomes; 4 n DNA: tetraploid chromosomes; DMEM: Dulbecco's modified Eagle's medium; EDTA: ethylenediaminetetraacetic acid; ELISA: enzyme-linked immunosorbent assay; FACS: fluorescence-activated cell sorting; FasL: Fas ligand; FCS: foetal calf serum; HU: hydroxyurea; ITS: insulin-transferrin-sodium selenite; NF-κB: nuclear factor-kappa-B; OD: optical density; PBS: phosphate-buffered saline; RA: rheumatoid arthritis; RASF: rheumatoid arthritis synovial fibroblast; RFU: relative fluorescence units; TNF: tumour necrosis factor; TRAIL: tumour necrosis factor-related apoptosis-inducing ligand.

## Competing interests

The authors declare that they have no competing interests.

## Authors' contributions

NP helped to design research, to perform research, and to analyse data and wrote the paper. MAP, CW, and TP helped to design research, to perform research, and to analyse data. IM helped to design research and to analyse data. SS, CF, KN, and CS helped to perform research. FvV helped to perform research and to analyse data. All authors read and approved the final manuscript.
